# *Mucor indicus* caused disseminated infection diagnosed by metagenomic next-generation sequencing in an acute myeloid leukemia patient: A case report

**DOI:** 10.3389/fcimb.2023.1089196

**Published:** 2023-02-07

**Authors:** Meixiao Shen, Qian Li, Zhaocheng Zeng, Dongxu Han, Xiansheng Luo

**Affiliations:** ^1^ Department of Hematology, Affiliated Haikou Hospital of Xiangya Medical College, Central South University, Haikou, China; ^2^ Genskey Medical Technology Co., Ltd, Beijing, China

**Keywords:** *mucor indicus*, disseminated mucormycosis, metagenomic next-generation sequencing, acute myeloid leukemia, liver abscess, allogeneic hematopoietic stem cell transplantation, case report

## Abstract

**Background:**

Mucormycosis commonly occurs in immunosuppressed patients with hematological diseases, which can be life-threatening. However, many cases are often misdiagnosed due to lack of specific clinical manifestations. Additionally, the traditional blood culture or serological testing, with a high false-negative rate, is time-consuming. Thus, precise and timely diagnosis of infections is essential for the clinical care of infected patients.

**Case presentation:**

We report a 29-year-old Chinese man with acute myeloid leukemia (AML) who developed febrile neutropenia after the first course of induction chemotherapy. He received empirical antibiotics, which did not relieve his symptoms. No pathogen was detected by traditional microbiologic assays, while *Mucor indicus* was identified by metagenomic next-generation sequencing (mNGS) in the blood specimen. Liposomal amphotericin B (LAmB) was used, resulting in the patient’s temperature returning to normal. A few days later, abdominal computed tomography (CT) scan showed multiple liver abscesses; fluorescence staining, histopathology, and mNGS identified the causative agent—*M. indicus.* Posaconazole was combined with LAmB as long-term antifungal treatment. Finally, the patient received allogeneic hematopoietic stem cell transplantation successfully after controlled infection. During follow-up 1 year after transplantation, the number of liver abscesses was reduced to one and remained stable.

**Conclusion:**

This report described the first case of an AML patient diagnosed with culture-negative disseminated infections caused by *M. indicus* leading to rare hepatic manifestations using mNGS of peripheral blood and liver biopsy. LAmB combined with posaconazole was given in time, resulting in a favorable outcome. mNGS is a new method that assists in detecting the probable pathogen and increases the accuracy of identifying an etiology.

## Introduction

Mucormycosis, caused by opportunistic pathogenic fungi that belong to the order Mucorales, commonly occurs in immunosuppressed patients with hematological diseases after chemotherapy or hematopoietic stem cell transplantation (HSCT) with a high mortality rate (Kontoyiannis et al., 2005; Roden et al., 2005; Petrikkos et al., 2012). The use of prophylactic and therapeutic drugs, such as glucocorticoids, immunosuppressants, and broad-spectrum antibiotics, has become an important pathogenic factor for mucormycosis infection in patients with acute leukemia, with pulmonary involvement being the most common presentation (Fadhel et al., 2019).

The gold standard for diagnosing mucormycosis is histologic findings and positive culture from blood or affected lesions. However, it is not feasible for vulnerable acute leukemia patients to obtain tissue biopsies. In addition, lack of regular septate in Mucorales species might contribute to fragile hypha; thus, it is difficult to culture *in vitro*, leading to delayed diagnosis (Spithoven et al., 2020). Metagenomic next-generation sequencing (mNGS), an unbiased approach capable of detecting causative pathogens contained in clinical specimens in a broad-spectrum manner, achieves accurate diagnosis and timely treatment for difficult-to-diagnose clinically moderate and severe infections (Chiu and Miller, 2019). Here, we presented the first case of an acute myeloid leukemia (AML) patient who experienced disseminated infections of *Mucor indicus* with rare hepatic manifestations, first detected by peripheral blood and liver biopsy through mNGS.

Liposomal amphotericin B (LAmB) and posaconazole are active against mucormycosis, but the required course of treatment for mucormycosis is not clear (Skiada et al., 2013; Liu et al., 2021; Wen et al., 2021). After a long-term follow-up of the case from the first chemotherapy to successful HSCT, for the first time, the total course of target antifungal agents was recorded in detail according to the patient’s condition and imaging manifestations, which provided a reference basis for the treatment of subsequent AML patients with mucormycosis.

## Case description

**Figure 1:**
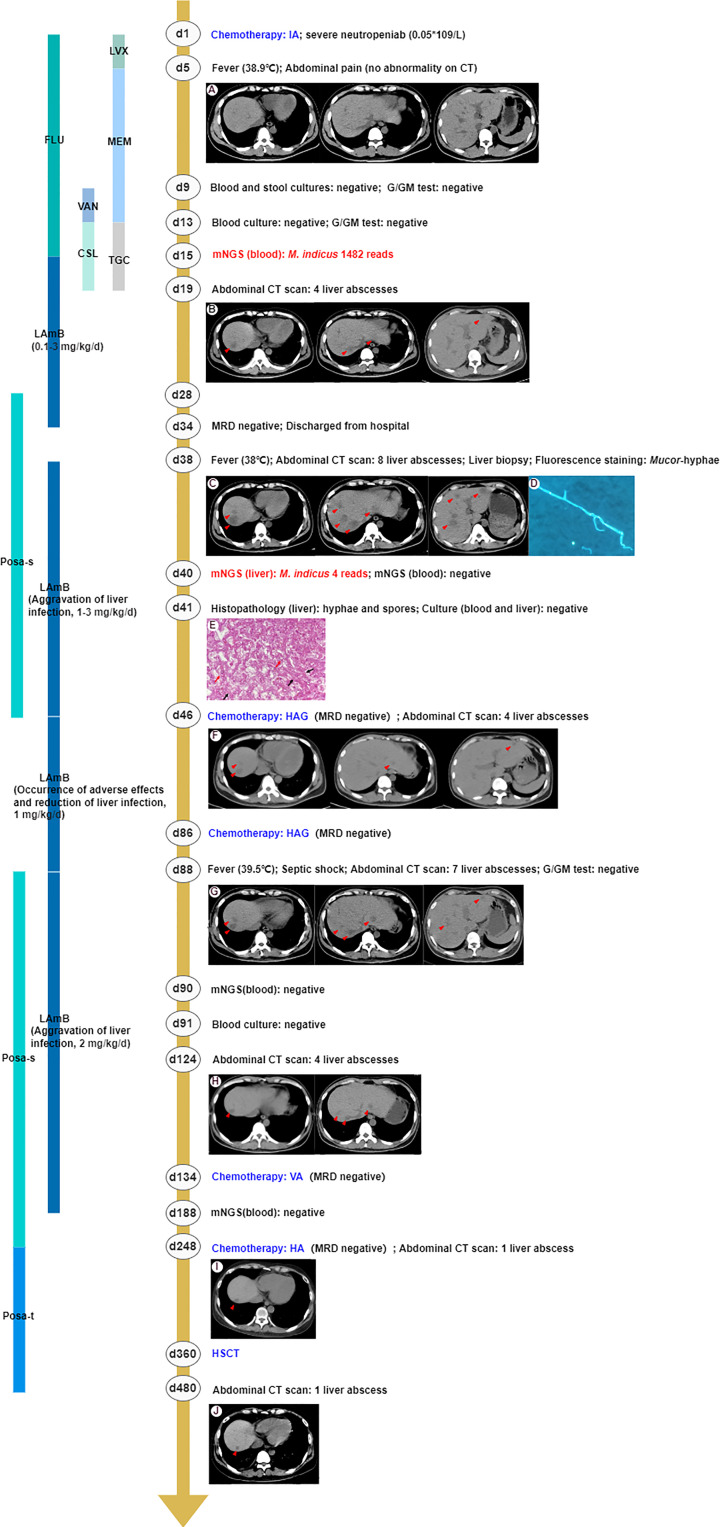
The clinical course of the patient (schematic). **(A)** The abdominal CT scan revealed no abnormalities. **(B)** The abdominal CT scan showed four low-attenuation liver lesions (marked in the red triangle). **(C)** Eight enlarged liver lesions were observed by abdominal CT. **(D)** Mucor-hyphae were found under fluorescence microscopy using CFW staining. **(E)** The histopathology of the liver showed non-septate, irregular, branched fungal hyphae (black arrow) and spores (red arrow). **(F)** The number of liver abscesses was reduced to four, revealed by abdominal CT scan, implying that antifungal therapy had been effective. **(G)** Seven liver abscesses were revealed by the abdominal CT scan, suggesting aggravation of liver infection. **(H)** The abdominal CT scan showed four liver lesions, implying that antifungal therapy had been effective. **(I, J)** The number of liver abscesses was reduced to one and remained stable. Chemotherapy IA, idarubicin and cytarabine; CT, computed tomography; G test, (1,3)-β-D-glucan test; GM test, galactomannan antigen test; mNGS, metagenomics next-generation sequencing; MDR, measurable residual disease; HAG, homoharringtonine, cytarabine and G-CSF; VA, venetoclax and azacitidine; HA, homoharringtonine and cytarabine; HSCT, hematopoietic stem cell transplantation; LVX, levofloxacin; FLU, fluconazole; MEM, meropenem; VAN, vancomycin; TGC, tigecycline; CSL, cefoperazone/sulbactam; LAmB, liposomal amphotericin B; Posa-s, posaconazole suspension; Posa-t, posaconazole tablet.

A 29-year-old man with intermediate-risk AML was treated with a standard chemotherapy regimen of 7 + 3 with idarubicin and cytarabine (day 1). Before receiving chemotherapy, the patient’s electrocardiogram, echocardiography, and abdominal ultrasonography did not show any obvious abnormality, except for a thoracic nodule shown in his chest computed tomography (CT) scan. The patient presented with severe neutropenia (0.05 × 10^9^/L after the first course of induction chemotherapy), and consequently, fluconazole (200 mg, daily, orally, days 1–15) and levofloxacin (500 mg, daily, orally, days 1–5) were empirically given as broad-spectrum antifungal and antibiotic prophylaxis, respectively. A timeline with relevant data and treatment from the patient in the course is shown in **Figure 1**. On day 5, the patient developed acute onset of fever to 38.9°C with abdominal pain, and his inflammation indicators increased. However, an abdominal CT scan revealed no abnormalities in the meantime. Possibly due to early empirical antimicrobial therapy, multiple clinical microbiologic assays including β-D-glucan and galactomannan (G and GM) tests, blood cultures, and stool cultures showed no pathogenic evidence. From day 5 to day 19, broad-spectrum antimicrobial coverage with meropenem combined with vancomycin and tigecycline combined with cefoperazone/sulbactam was empirically administrated for poor immunity, suggestive of potential infection of pathogens. However, there was no significant relief from his symptoms. Therefore, the patient’s peripheral blood samples were sent for mNGS to improve the etiological examination on day 13 (Jing et al., 2021). Within 48 h after receipt of the samples, the presence of *M. indicus* was examined by mNGS analysis with 1,482 reads. The anti-infection regimen was changed to Liposomal amphotericin B (LAmB) immediately (escalated from 0.1 mg/kg to 3 mg/kg daily, intravenous injection, days 15–34) with subsequent defervescence. On day 19, an abdominal CT scan showed four low-attenuation liver lesions caused by an invasive microorganism (Khim et al., 2019). However, the liver biopsy was not performed immediately owing to thrombocytopenia (20 × 10^9^/L) and leukopenia (0.1 × 10^9^/L).

On day 34, the patient achieved complete remission with a negative measurable residual disease (MRD) result and then was discharged from the hospital with instructions to continue his routine treatment with daily oral posaconazole suspension (200 mg, qid, po, days 28–46). After 4 days, the patient was readmitted to the hospital for the first course of consolidation chemotherapy. On admission, due to the enlarged liver lesions observed by abdominal CT, suggesting aggravation of liver infection, we obtained lesion specimens through an ultrasound-guided liver biopsy for pathogenic examinations and administered LAmB (escalated from 1 mg/kg to 3 mg/kg, daily, intravenous injection) as the antifungal agent. Fluorescence staining observed ribbon-like, folded hyphae. No pathogen was cultured from liver biopsy tissues, but histology revealed non-septate, irregular, branched fungal hyphae and spores. Meanwhile, mNGS detected sequence reads corresponding to *M. indicus* (4 reads), confirming that mucormycosis also occurred in the liver. The diagnoses of disseminated infections caused by *M. indicus*, including liver and bloodstream infections, should be clear at this point.

Patients received two homoharringtonine, cytarabine, and G-CSF (HAG) consolidation chemotherapy sessions on days 46 and 86, respectively, during which intravenous LAmB was decreased to 1 mg/kg/day due to reduction of liver abscesses to four and adverse effects of LAmB (severe gastrointestinal reaction, hypokalemia, and hepatic and renal insufficiency). The patient suffered from hypotension (80/42 mm Hg) and fever (39.5°C) caused by septic shock and stopped chemotherapy on the 88th day and received meropenem and linezolid administered for 1 week. Coincident with sending peripheral blood samples for mNGS and culture in an attempt to investigate possible etiology, posaconazole suspension (200 mg, qid, po, days 88–248) and a higher dose of LAmB (2 mg/kg, daily, intravenous injection) were used on day 88 for three additional liver abscesses shown on the abdominal CT scan. After 2 days, the mNGS of peripheral blood showed a negative result, which was consistent with the blood culture result 1 day later. However, the liver abscesses were reduced to four on day 124, implying that antifungal therapy had been effective. Given the inactive hepatic abscesses and leukemia treatment, the patient received venetoclax and azacitidine (VA) and homoharringtonine and cytarabine (HA) chemotherapy on days 134 and 248, respectively. Based on the negative mNGS results of peripheral blood, stable liver lesions, and continuous hepatic and renal insufficiency (AST 55 U/L, ALT 45 U/L, blood urea nitrogen 9.9 mmol/L, creatinine 171 μmol/L, GFR 42 ml/min/1.73 m^2^), the patient stopped using LAmB intravenously on day 188 but continued with oral posaconazole (changed to tablet, days 248–480) as against fungal infection.

On day 360, the patient received peripheral blood stem cells from an unrelated donor. The chimerism rate was 99.5% at day 30 post-HSCT, which meant complete donor chimerism. The total course of LAmB was 8 months, and the cumulative dose was 20.5 g. The entire course of oral posaconazole was almost 1 year, consisting of the whole transplantation period and 4 months post-HSCT. Until now (day 780), the number of liver abscesses remained at one and remained stable. The major problem after HSCT was renal insufficiency (blood urea nitrogen 8.3 mmol/L, creatinine 168 μmol/L, GFR 43 ml/min/1.73 m^2^) and grade 1 skin graft-versus-host disease. The liver function returned to normal.

## Discussion and conclusions

Mucormycosis, caused by opportunistic pathogenic fungi that belong to the order Mucorales, is a difficult-to-diagnose rare disease with high mortality that commonly occurs in patients with impaired immune status, particularly those with diabetes mellitus, hematological malignancy, and neutropenia (Dong et al., 2022). According to literature review, this is the first reported patient with AML who developed disseminated infection caused by *M. indicus* (**Supplementary Table S1**). The most frequent clinical presentations of mucormycosis are pulmonary, sinusitis, and cutaneous (Roden et al., 2005). The hepatic manifestations presented in our case are rare and may arise from hematogenous dissemination from an infected vascular catheter (Weng et al., 1998).

Due to the rapidly progressive infection, mucormycosis requires urgent diagnosis and intervention to reduce mortality. Culture is highly recommended to confirm the diagnosis of mucormycosis in tissues, but it is time-consuming, with a high false-negative rate. Microscopic identification is often used clinically for rapid presumptive diagnosis of mucormycosis, but this method does not allow identification to the genus or species level, which is important to guide antifungal therapy, because of the different clinical picture depending on the species (Abela et al., 2013). Clinical mNGS, an unbiased approach capable of detecting causative pathogens at the species level, has fundamentally changed the management of mucormycosis, which can achieve accurate diagnosis and timely treatment for difficult-to-diagnose fatal infections. As far as we know, this is the first report wherein mNGS can detect *M. indicus* from peripheral blood and liver biopsy tissue, with culture and serological testing yielding negative results (**Supplementary Table 1**). Moreover, the low sequence reads of *M. indicus* detected in liver biopsy tissue were caused by formalin-fixed, paraffin-embedded tissue samples we used for sequencing, as formalin damages DNA (Cornely et al., 2019).

Global guidelines for the diagnosis and management of mucormycosis strongly support early and complete surgical treatment of mucormycosis, in addition to systemic antifungal therapy (Cornely et al., 2019). Considering the deteriorating systemic condition and multiple infected lesions in this case, surgical debridement was not possible. We treated the patient with an empirical combination of LAmB and posaconazole, which is regarded as the most common treatment for mucormycosis. However, the duration of treatment required to treat mucormycosis is not clear (Cornely et al., 2019). In this case, continuous medication adjustments were made based on the patient’s condition, medication side effects, and imaging manifestations until substantial radiographical improvement of the liver. The patient then successfully underwent HSCT.

In conclusion, we diagnosed disseminated infections caused by *M. indicus* in an AML patient with rare hepatic manifestations using mNGS. LAmB combined with posaconazole was given, and the patient received allogeneic HSCT successfully after controlled infection. This report suggests that mNGS is a new method that assists in finding the causative pathogens with no other specific diagnostic basis and in helping to cure the patient.

## Data availability statement

The datasets presented in this study can be found in online repositories. The names of the repository/repositories and accession number(s) can be found below: https://ngdc.cncb.ac.cn/, PRJCA012925.

## Ethics statement

Ethical review and approval was not required for the study on human participants in accordance with the local legislation and institutional requirements. The patients/participants provided their written informed consent to participate in this study. Written informed consent was obtained from the individual(s) for the publication of any potentially identifiable images or data included in this article.

## Author contributions

MS designed the study, analyzed and interpreted the patient data, and wrote the manuscript. QL contributed to data collection and analysis, and manuscript writing. XL performed data collection, manuscript review, and revision. DH contributed to manuscript review. ZZ provided data acquisition, analysis, and interpretation. All authors read and approved the final manuscript.
