# Supplementary material for: Mucor indicus caused disseminated infection diagnosed by metagenomic next-generation sequencing in an acute myeloid leukemia patient: A case report
Source: Front Cell Infect Microbiol. 2023 Feb 7;13:1089196. doi: 10.3389/fcimb.2023.1089196 (PMC9941182; doi:10.3389/fcimb.2023.1089196)
Supplement: Supplementary file 1 [file Table_1.docx]

**Table S1**. **Summary of the Characteristics Among 13 cases with *M. indicus* infections.** M, Male; F, Female, NA, Not available; LBL, Lymphoblastic leukemia; MDS, Myelodysplastic syndrome; GVHD, Graft versus host disease; ALL, Acute lymphocyte leukemia; AmB, Amphotericin B desoxycholate; ABLC, Amphotericin B lipid complex; VRC, voriconazole; PCR, Polymerase Chain Reaction.

| **Reference** | **Age /sex** | **Underlying disease** | **Immunosuppression or chemotherapy** | **Site of infection** | **Diagnosis method** | **Antifungal before diagnosis** | **Surgical** | **Antifungal therapy** | **Outcome** |
| --- | --- | --- | --- | --- | --- | --- | --- | --- | --- |
| Douvin et al., 1975 [10] | 66/M | None | NA | Gastric ulcer | Pathology | NA | Yes | AmB | Recovery |
| Borg et al., 1990 [11] | 27/F | Acute T- LBL | Yes | Appendices, liver | Microscopy, culture | Ketoconazole | NA | AmB, 5-flucytosine | Died |
| Oliver et al., 1996 [12] | 39/M | MDS, HSCT, acute GVHD | Yes | Liver, diaphragm, abdominal wall | Microscopy, culture, PCR | NA | Yes | AmB, switched to ABLC and flucytosine | Recovery |
| Soble, 2001 [13] | 56/F | None | NA | Vagina | Microscopy, culture | Multiple azoles (topical and systemic) | NA | Topical AmB | Recovery |
| Mata-essayag et al., 2001 [14] | 82/F | Cutaneous trauma | NA | Necrotic tissue of knee | Microscopy, culture | NA | Yes | AmB, fluconazole | Recovery |
| Aboltins et al., 2006 [15] | 34/M | None | NA | Abdomen, blood | Culture, PCR | NA | No | ABLC, then L-AmB | Recovery |
| Deja et al., 2006 [16] | 48/M | Acute head injury | NA | Stomach, ileocecal valve, colon | Culture, PCR, pathology | NA | Yes | LAmB, and local AMB via gastric tube | Recovery |
| Repentigny et al., 2008 [17] | 6-month-old/F | Assist device heart implant | NA | Ascending aorta, pericardium, urine | Microscopy, culture, PCR, pathology | NA | Yes | No | Died |
| Satoko et al., 2013 [18] | 62/F | Acute LBL, HSCT | Yes | Colon, liver | Pathology | VRC | Yes | VRC switched to AmB | Died |
| Luo et al., 2014 [19] | 58/M | Tibial injury | No | Pretibial area | Microscopy, culture, PCR, pathology | Fluconazole | Yes | AmB, then oral itraconazole | Recovery |
| Bloch et al., 2018 [20] | 4/M | Pre-B-ALL | Yes | Groin abscesses, lymph nodes | Microscopy, culture, PCR, pathology | VRC | Yes | LAmB, then oral Posaconazole | Recovery |
| Uchida et al., 2019 [21] | 82/M | Adult-onset Still disease, diabetes | Yes | Stomach | Culture, PCR, pathology | NA | No | LAmB | Died |
| Shen et al.,2022 (this case) | 29/M | Acute myeloid leukemia | Yes | Blood, liver | Microscopy, pathology, mNGS | Fluconazole | No | LAmB and oral Posaconazole | Recovery |
